# Discrepancies in Embryonic Staging: Towards a Gold Standard

**DOI:** 10.3390/life13051084

**Published:** 2023-04-26

**Authors:** Sander Flierman, Melanie Tijsterman, Melek Rousian, Bernadette S. de Bakker

**Affiliations:** 1 Department of Obstetrics and Gynecology, Amsterdam UMC Location University of Amsterdam, 1105 AZ Amsterdam, The Netherlands; 2 Department of Obstetrics and Gynecology, Erasmus MC-University Medical Center Rotterdam, 3015 GD Rotterdam, The Netherlands; 3 Amsterdam Reproduction and Development Research Institute, 1100 DD Amsterdam, The Netherlands; 4 Sophia Children’s Hospital, Department of Pediatric Surgery, Erasmus MC-University Medical Center Rotterdam, 3015 GD Rotterdam, The Netherlands

**Keywords:** Carnegie staging system, developmental horizons, embryology, embryonic staging, embryonic development

## Abstract

For over half a century, the Carnegie staging system has been used for the unification of chronology in human embryo development. Despite the system’s establishment as a “universal” system, Carnegie staging reference charts display a high level of variation. To establish a clear understanding for embryologists and medical professionals, we aimed to answer the following question: does a gold standard of Carnegie staging exist, and if so, which set of proposed measures/characteristics would it include? We aimed to provide a clear overview of the variations in published Carnegie staging charts to compare and analyze these differences and propose potential explanatory factors. A review of the literature was performed, wherein 113 publications were identified and screened based on title and abstract. Twenty-six relevant titles and abstracts were assessed based on the full text. After exclusion, nine remaining publications were critically appraised. We observed consistent variations in data sets, especially regarding embryonic age, varying as large as 11 days between publications. Similarly, for embryonic length, large variations were present. These large variations are possibly attributable to sampling differences, developing technology, and differences in data collection. Based on the reviewed studies, we propose the Carnegie staging system of Prof. Hill as a gold standard amongst the available data sets in the literature.

## 1. Introduction

**Figure 1:**
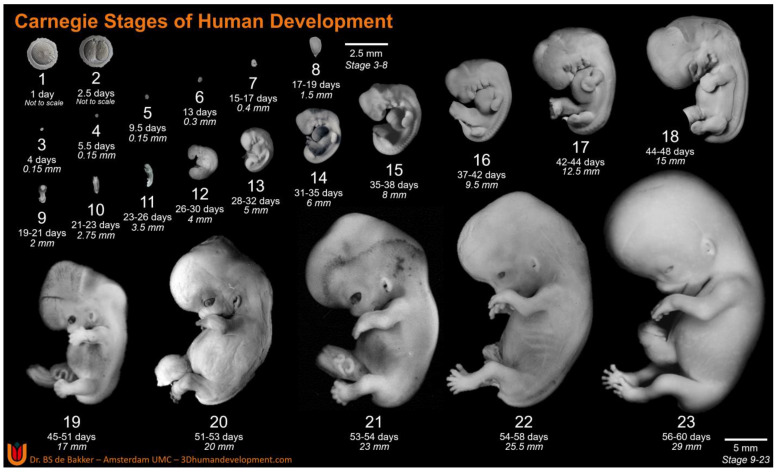
Carnegie Stages of Human Development. Carnegie Stages 3–10, dorsal view; Stage 11 onwards, left lateral view. Ages and lengths derived from O’Rahilly (1987) for Stages 1–6 and Hill (2007) for Stages 7 onwards [4,5]. CS1 and 2 are imaged by Dr. Mastenbroek, Amsterdam UMC (personal communication with permission), CS3–8 are 3D reconstructions based on histological sections from the Carnegie collection (3Dhumandevelopment.com, accessed date 1 March 2023) [6]. Original figures of CS9–22 are derived from the Carnegie collection, National Museum of Health and Medicine, Silver Spring, MD, USA, and CS23 is adapted with permission from Hill (2018) [7], copyright 2023, John Wiley and Sons. Exact specimen numbers from the Carnegie collection: CS3–8794, CS4–0610, CS5–8020, CS6–7801, CS7–8752, CS8–8671, CS9-H712, CS10–6330, CS11–6344, CS12-8505A, CS13–0836, CS14–8314, CS15–3512, CS16–6517, CS17–6521, CS18–6524, CS19–2114, CS20–0462, CS21–4090, CS22–0895.

Derived from the Greek “*embryon*”, embryology is the understanding of how our bodies came into being. More specifically, it is the branch of biology that studies the formation, growth, and development of an embryo from a fertilized egg [1]. Findings within this field have helped to develop our understanding of congenital abnormalities and their respective solutions. From as early as 1969, the importance of establishing a chronological timeline within human embryonic development was understood as “The need for standardized stages in the embryonic development of various organisms for the purpose of accurate description of normal development and for utilization in experimental work has long been recognized” [2]. As such, a morphological scheme was devised to provide a standardized and unified staging system of embryonic development. Composed of 23 unique and detailed stages (Figure 1), the Carnegie staging system helps to distinguish the key structural developments of the vertebrate embryo [3]. For humans, this staging system provides an in-depth coverage of the first 60 days within embryonic development, otherwise known as the embryonic period.

Despite its use as a universal staging system for ex vivo human embryos, the literature regarding the distinctions between the Carnegie stages is inconsistent and convoluted, with leading researchers supplying differing understandings and data on the respective internal and external embryonic features allocated to each individual stage. Furthermore, no verified explanation for these discrepancies amongst established researchers could be located, highlighting a prominent gap in the research and understanding regarding the most established embryological staging system.

Therefore, the goal of this research can be broken down into several aims, the first of which was to provide a clear overview of the variations in commonly available Carnegie staging charts, wherein the differing data are compared and analyzed to establish a clear overview for embryologists or other medical professionals by means of a review of the literature. Secondly, we aimed to explain the presence of these differences by evaluating how these data were collected (e.g., post ovulatory days). In doing so, we aimed to research whether a gold standard for Carnegie staging charts exists, and if so, which chart it would be. Subsequently, we aimed to better standardize the staging system used across the field of embryology.

## 2. Background: Historical Beginnings of Embryonic Staging

Although embryonic staging was introduced as early as the 1800s, the use of such a staging system on humans was only employed early in the 20th century. Founded by Franklin P. Mall, the Carnegie collection is composed of numerous sectioned and serially conducted ex vivo human embryos. With its first designated human embryo cataloged in 1887, this detailed and quintessential collection would subsequently grow, lending valuable knowledge to its directors and international researchers alike. Furthermore, detailed reconstructions and elaborate drawings based on this collection were published and applied within academic writing from 1890 onwards, paving the way for detailed analyses and deeper understanding in a previously inaccessible scientific field.

Named after this detailed collection of human embryos, the Carnegie stages are based on a combination of several embryonic features. Beyond morphological features, the stages include age ranges, number of somites present, and embryonic length (mm). However, these factors are less heavily weighed against morphological changes due to the higher variability and how single size values or somite levels may span across multiple stages [4].

The notion of developmental stages was first introduced in 1914 by Franklin P. Mall [8], who categorized 266 human embryos, splitting them amongst 14 separate “stages” during his time as director of the Carnegie collection. Shortly thereafter, Mall’s position and proposition would be replaced by George L. Streeter, who further refined Mall’s 14 embryonic stages into 23 “developmental horizons” [9]. The term “horizons”, borrowed from archaeology and geology, was utilized by Streeter to stress the ever-increasing complexity of developing embryos. Despite initially planning on composing twenty-five distinct age groups, Streeter subsequently concluded that 23 stages could effectively encompass the embryonic period [9]. This use of 23 stages (Figure 1) [10] was applied, as “each stage is merely an arbitrary cut section through the time-axis of the life of an organism” [11]. Upon further research, these developmental horizons were better described and distinguished by Ronan O’Rahilly and his wife, Fabiola Müller, in 1987, who retained the use of 23 distinct stages, but proposed the term “stages” in place of “horizons”, due to its simpler and more comprehensible nature [4]. During his time serving as the director of the Carnegie collection (since 1973), O’Rahilly’s work on staging went through several iterations, becoming the first widely recognized staging system for human embryos. Since then, no major alterations have been made, as alternative systems and terminology for embryonic staging have never maintained a foothold within research and have ultimately been rendered obsolete.

## 3. Embryonic Age

**Figure 2:**
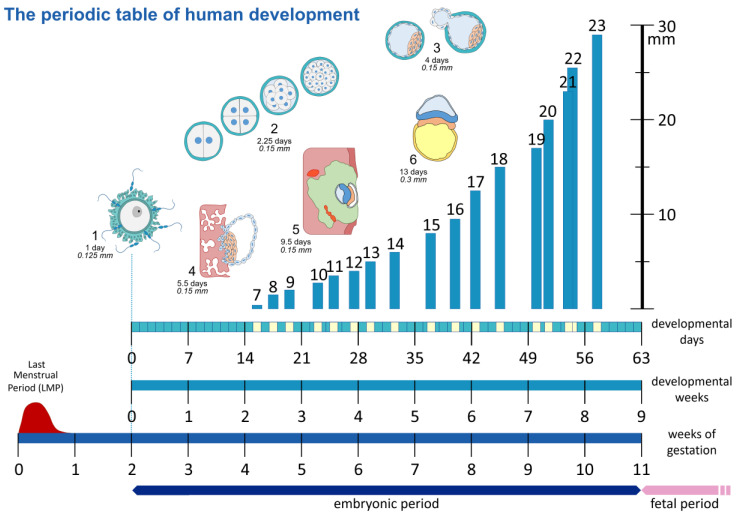
The “periodic table” of human development. Showcasing the different methods to date a pregnancy, as gestational age based on the last menstrual period (LMP), the postovulatory or embryonic age as developmental days or weeks, counted from the time of fertilization. Schematic representation of Carnegie Stages 1–6 (not to scale). Carnegie Stages 7–23 are plotted against length in mm on the *y*-axis, and developmental days are on the *x*-axis.

The term “embryonic age” and what exactly it entails has always been a point of contention amongst naturalists and embryologists alike and has been laced with ambiguity and disagreements. To provide clarity, this section is included to situate current academic understanding and shine a light on areas of confusion. A range of challenges exist in attempting to determine the age of an embryo, but most importantly amongst them is the lack of a precise timing or indicator as to when fertilization occurs [12]. Hence, two primarily utilized measurements should be highlighted. The first of these measurements is gestational age. Gestational age can be defined as a measure of the age of a pregnancy that is taken from the beginning of the woman’s last menstrual period (LMP). In general, the starting point of this measure is approximately two weeks before the actual fertilization. In contrast to this is the developmental or postovulatory age. This measure represents the actual age of the embryo by utilizing the time of fertilization as a starting point, as showcased within Figure 2. Due to this, the difference between these two measures is approximately two whole weeks, and therefore the establishment and clarification of which system is being applied with regards to the age of the embryo is essential.

## 4. Embryonic Length

From as early as 1749, the utilization of embryonic length to determine age (which would, in turn, be translatable to Carnegie stage) was attempted [13,14]. Interestingly, over the course of the last two centuries, embryonic staging through the use of embryonic length has been rendered much more precise, and due to current technological advancements, embryonic length can now serve as a workable estimate of the embryos’ respective Carnegie stage. However, within the clinical setting, the notion of what axis of the embryo should be measured showcases no singular consensus. With possibilities such as head circumference (HC), biparietal diameter (BPD), and brain length, it remains inconclusive which measurement can most accurately stage embryos. This dilemma is further complicated, as certain measurements only become feasible further into development. 

Amongst the available measurements, the crown–rump length (CRL) appears to be most frequently used. Defined as the distance between the top of the head (crown) and the bottom of the buttocks (rump), the CRL can be measured through the use of an ultrasound and has showcased exceptional use in calculating the gestational age of the embryo [15]. Henceforth, when embryonic length is mentioned in the paper, it refers to CRL, in line with its prominent use in papers such as O’Rahilly, Hill, and Nishimura et al. [4,5,16].

## 5. Carnegie Stages: Academic Discrepancies and Nonuniformity

When evaluating the Carnegie staging system, Streeter’s work from 1951 is frequently viewed as the foundation upon which current understanding was built [9]. However, across the relevant international literature, variations within the internal and external embryonic features for each stage are present, such as inconsistent ages (days) and varying somite numbers [16,17,18]. Due to this individual variation across scholarly publications, staging criteria such as the mean dates for each stage are not uniform across the literature. This inevitably threatens the “universal” purpose of the staging system, as the system is no longer standardized. Therefore, it remains difficult for professionals in the field to decide which Carnegie staging chart should be consulted.

In modern research, O’Rahilly and Müller’s revision of Streeter’s work is more widespread, despite the significant differences from Streeter’s publication, in both morphological and non-morphological features [4,9]. Yamada et al. helps to clearly showcase these differences and highlights the relationship between embryonic ages in respective Carnegie stages from various researchers, including O’Rahilly and Streeter [19]. Alongside these publications, several other researchers have brought forth their individual Carnegie staging charts, e.g., Nishimura et al. proposing values that vary as far as 2–3 days off from the mean dates of O’Rahilly’s values [4,16,20]. Similarly, such variation can be seen within publications from Jirásek [17], Hill [5], Harkness [21], the Human Developmental Biology Resource [22], and the *Heirloom Collection* [23]. To further complicate the selection process for Carnegie charts, prominent researchers such as O’Rahilly have been shown to further build upon their previous work, releasing updated or revised values of their prior Carnegie stages. One key example of this would be O’Rahilly’s revision, which was published in 2010, and altered numerous values and criteria [24]. However, the relevance of this aforementioned revision is questionable, with numerous researchers within the field choosing to refer to the 1987 iteration instead, due to its established credibility.

Despite a scarcity of embryology resources that are available to the general public, M. Hill has worked on making this information more accessible to the public through the use of the *Embryology Education and Research* website [7]. Similarly, the values provided within this website differ from the values present within O’Rahilly’s published work from 1987. Due to its intended use as an educational resource, understanding the underlying reasons for such discrepancies is of the utmost importance.

### The Importance of Concise Embryonic Staging Systems

With regards to relevance and rationale, the need for precision and clarity within the medical field should be well understood. Within the field of embryology, it is important to differentiate the different developmental stages to identify developmental anomalies. This would allow medical professionals to more actively notice embryonic complications and reduce the uncertainty within an already highly variable field. Regarding its relevance, the standardization of these Carnegie stages would be primarily noticeable within maternal and prenatal care. Within such a clinical field, accurate estimation of developmental age is of utmost importance, as an incorrect estimation can have short- and long-term consequences for both the mother and unborn child (e.g., iatrogenic labor at a premature age instead of a term age). Within the Netherlands, the screening procedure employed (e.g., NIPT-test and 13- and 20-week anomaly ultrasound scan) is almost exclusively based upon an accurate gestational age estimation. Gestational age estimation in pregnancy includes a pregnancy dating ultrasound scan, purely based on the CRL measurement of the developing fetus between the 10th and 12th week of pregnancy [25,26]. Knowing that the improved quality of ultrasound machines allows for earlier (3D) ultrasound examinations, including volumetric measurements of the embryo [27], the Carnegie staging system should serve as a consistent and reliable source of values that can be consulted amidst confusion. Therefore, formulating a cohesive understanding and a consistent set of values for the “universal” staging system of embryos is essential, with its effects cascading to expecting mothers and clinicians alike.

## 6. Methods

**Figure 3:**
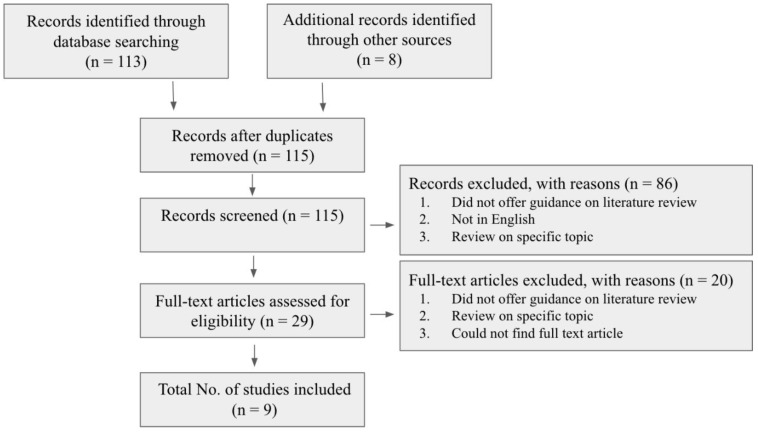
Flow diagram showcasing the literature review process.

For this review, the methodology was split into two sections. The first section was a review of the literature that focused on a few established academic works, each of which proposed a differing set of data/characteristics for the individual stages of the Carnegie system. A review of the literature was utilized to allow us to compare and contrast existing differences across the slightly varied Carnegie charts (Figure 3). For this process, works from the English scientific literature were included. These covered the topic of embryonic growth or embryonic staging but did not extend to the fetal period. For the literature search, no restrictions were set on the publication date, as within the field of embryology, the early literature is still highly relevant and applicable. Sources were selected using the following keywords: Carnegie system, embryonic growth, developmental horizons, embryology, and embryonic stages. These sources were then screened initially according to their title and abstract, and, subsequently, the full-text articles were skimmed to further evaluate the quality and eligibility of the studies. This was performed to investigate the differing charts and data.

Secondly, we investigated which of the aforementioned charts is most frequently applied in the embryology literature. To research this, another review of the literature was conducted with similar parameters and criteria. These sources were subsequently screened to achieve a clear overview of the most frequently applied Carnegie staging values. Both of these reviews of the literature were carried out by one reviewer, under the supervision of experienced embryologists. All of this was carried out with the aim of reaching a consensus on what staging method should be used as the gold standard of embryological staging to ascertain the most agreed-upon and universal set of values that can be consulted with regards to gestational age, embryonic length, and somite numbers.

## 7. Results

**Figure 4:**
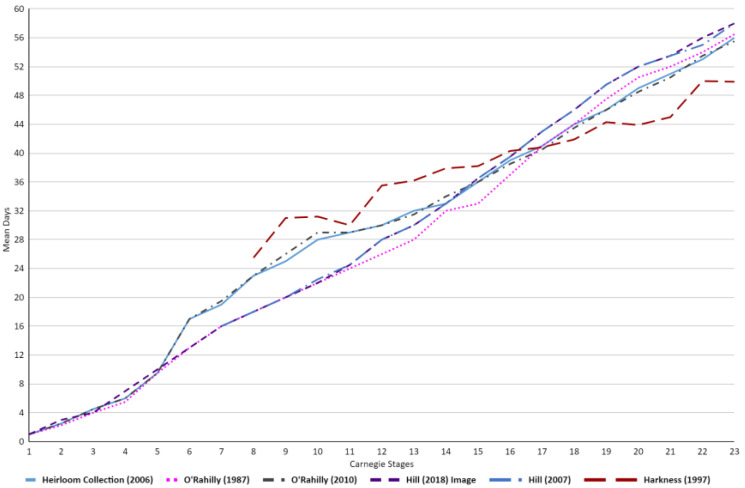
Graph demonstrating the differences in embryonic age (mean days), across the embryonic literature [4,5,18,21,23,24], in relation to Carnegie stages. The first 6 stages of Hill were not present within his publication and as such are not showcased within the graph [5,18]. Similarly for Harkness [21], the first 7 stages are not included.

Within Table 1 and Figure 4, stage 1–5 embryos showcase approximately equal mean days across the various academic publications. However, as early as stage 6, substantial differences can be observed between the studies. The presence of a steep increase in mean days can be seen within the Heirloom Collection and O’Rahilly [23,24]. These higher mean day values compared to the other studies remain present throughout stages 6–13, after which a more uniform data set can be observed across the studies once again. Furthermore, Harkness also showcases a much higher mean days value at stage 8 and maintains this higher average till approximately stage 16–17, wherein it falls below the average trend of the other academic publications [21].

Interestingly, both of Hill’s publications [5,18] showcased a higher terminal mean days for Carnegie stage 23 embryos (~2–3 days higher than other publications). Alongside this, the mean days published by Hill underwent minute changes or revisions between 2007 and 2018, with the biggest change being observed for Stage 22 (1 day difference) [5,18]. Contrary to this would be O’Rahilly’s publications, which displayed widely revised mean days, with differences as large as 7 days (Stage 10) between his 1987 and 2010 paper [4,24].

**Figure 5:**
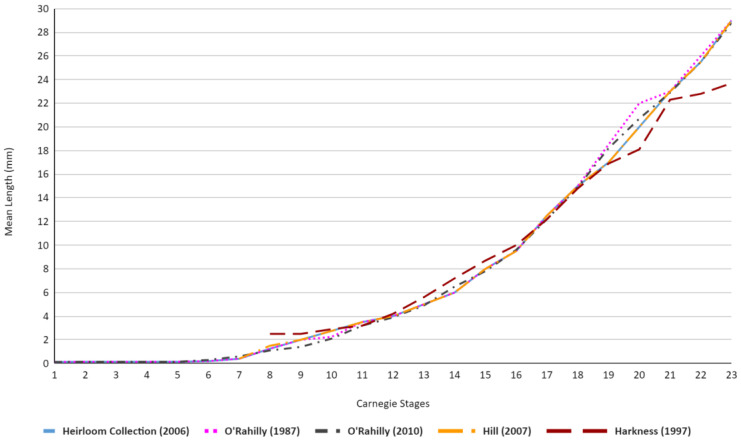
Graph demonstrating the differences in embryonic length (mm) across the embryonic literature [4,5,21,23,24] in relation to Carnegie stages. The first 6 stages of Hill were not present within his publication and, as such, are not showcased within the graph [5]. Similarly, for Harkness [21], the first 7 stages are not included.

Table 2 and Figure 5 showcase similar trends, although a majority of the results display more uniform results, with the exception of Stages 18–21 within O’Rahilly’s [4,24] values, which vary up to 2 mm from the majority of other academic publications. Similar to Figure 4, Harkness’ values also showcase a higher mean length value at stage 8, although this higher average only remains present in stages 8 through 10, wherein it falls in line with the average trend of the other academic publications before subsequently falling below the average trend once more for stages 19–23 [21]. 

## 8. Discussion

When comparing the embryonic ages and lengths within the current literature, we observed a wholesomely non-uniform set of values. The aim of the current study was to provide an overview of variations in Carnegie staging charts in the available literature and to clarify if a gold standard of Carnegie staging could be identified through the comparison of various reputable academic publications and their respective data sets and differences (Table 3). Based on Figure 4 and Figure 5 presented here, we can conclude that the “universal” staging system is peppered with discrepancies and ambiguity and remains inconclusive regarding which of the studies should be consulted. Although the cause behind these variances is not fully understood, we sought to propose a set of factors that may have played a role in this dissimilarity to better ascertain which publication should be consulted for the most accurate staging.

### 8.1. Staging Differences: A Matter of Sampling?

Within embryological research, the acquisition of human embryo samples has been an ethical challenge throughout the history of the field, due to rigorous guidelines and regulations. Subsequently, across the various studies analyzed within this review, the samples and sampling methods applied differ greatly. Within both his 1987 publication and his 2010 revision, O’Rahilly utilized embryos from the Carnegie collection, composed of a mixture of human histology and fixed specimens [4,24]. At the time of his initial publication, the Carnegie collection served as the most reputable collection of human embryos, contributing to the credibility of his study.

However, Hill’s recent publication in 2007 utilized a wider set of samples [5] and, instead, analyzed embryonic samples from both the Carnegie collection and the Kyoto collection in Japan (details on these collections shown within Table 4). Although the use of a multi-collection approach was not available to O’Rahilly at the time, modern web resources such as the Human Embryology website [28] have enabled researchers to expand upon their sample sizes. Aside from the use of pre-existing collections, certain authors, such as Harkness, opt to utilize a new collection of embryos [21], ascertained through abortions regarding embryos which have undergone less than 9 weeks of gestation, and were referred to the researchers by local family planning services and general practitioners. Although it is impossible to discern to what extent this difference in sampling technique might affect embryonic age and length, it is well within reasoning to attribute some of these differences in values to sampling.

**Table 3:** Characteristics per Carnegie Stage.

Carnegie Stage	P.O. Days	Size (mm)	Characteristics	Carnegie Stage	P.O. Days	Size (mm)	Characteristics
1	1	0.125	Unicellular	13	28	5	Lower limb buds appear as bulges Caudal neuropore closed Lens placodes Otic vesicles Left and right lung buds discernable Septum primum and foramen primum
2	1.5–3	0.15	More than one cell presentNo blastocystic cavity present	14	32	6	Longer upper limbs Lower limbs clearly visible Nasal pits Optic cups
3	4	0.15	Blastocyst	15	33	8	Handplate Lower limbs elongate Future cerebral hemispheres distinct Foramen secundum in the heart
4	5–6	0.15	Zona pellucida dissolved Blastocyst attachment to uterine epithelium	16	37	9.5	Slight rotation upper limbs Footplate Pigment in the retina
5	7–12	0.15	Solid trophoblast Trophoblastic lacunae Primary umbilical vesicle Mesoblastic crests Lacunar vascular circle	17	41	12.5	Digital rays in hand plate Slight rotation of lower limbs Cerebral vesicles clearly visible Semilunar cusps visible in the heart Foramen primum obliterated
6	13	0.2	Chorionic villi Primitive streak Secondary umbilical vesicle Cloacal membrane	18	44	15	Longer and straighter trunk, toe rays Scalloping hand plate, start digits 4th ventricle larger than lateral ventr. Elbow region visible Membran. region interventr. septum Septum secundum
7	16	0.4	Cranial prolongation primitive streak (notoch. process) Primitive node Secondary villi Cloacal membrane Allantoic diverticulum	19	47.5	18.5	Elongation and straightening of trunk Upper limbs slightly bent in elbow Limbs extend ventrally Hands far apart, short fingers Midgut herniation
8	18	1.25	Primitive node Notochordal process Prechordal plate Primitive pit Notochordal canal	20	50.5	22	Longer upper limbs, bent in elbow, hands slightly flexed Toes separated 4th ventr. still larger than lateral ventr. Scalp vascular plexus visible
9	20	2	Head fold Somite pairs	21	52	23	Hands and feet turned inward Longer fingers Toes distinct but webbed Bending of knees, toes may touch Trunk straight and longer Stubby tail visible
10	22	2.25	Neural tube closing Looped heart tube	22	54	26	Eyelids visible Fingers may overlap Lower limbs rotated, touching feet Very straight trunk 4th ventricle smaller than lateral ventr. Hemispheres recognizable
11	24	3.5	Rostral neuropore closing Otic placodes Optic vesicles 1st and 2nd phar. arches Meson. duct and tubules Sinus venosus	23	56.5	29	Rounded head Limbs increased in length Rotation of lower limbs Forearm ascends to shoulder level Scalp vascular plexus at vertex
12	26	4	Rostral neuropore closed Caudal neuropore closing Upper limb buds 3rd pharyngeal arch Otic pits Lung bud Interventricular septum formation	*Characteristics of Stages 1–8 were taken from O’Rahilly’s study (1987) [4], and the characteristics of Stages 9–23 were acquired through a combination of sources, including the HDBR atlas, O’Rahilly (1987), Hill (2007), and Pietersma (2023) [4,5,22,29]. P.O days and embryonic size data utilized within the table were taken from O’Rahilly’s study (1987)* [4].			

This substantial difference might also be attributable to the fact that O’Rahilly utilized 310 embryos that were deemed of “good/excellent” condition. Throughout their research, both Hill and O’Rahilly avoided strictly “abnormal” specimens. In doing so, values and data sets obtained would therefore be more concise and reliable, with less outliers. This would, in turn, allow the results of the study to be more generalizable and applicable to healthy embryos. Contrary to this, Harkness utilized any embryo that was accessible, applying the exclusion criteria of mothers with evidence of multifetal pregnancies, history of serious medical disorders, and those aged less than 17 years [21]. However, as our knowledge within embryology has changed, so has our perception on the normality of specimens, and more and more embryos that were previously deemed normal have showcased signs of newly discovered abnormality. This shift in perception could play a role in the differences, as the embryos upon which these various studies were conducted may ultimately not fall under the same modern-day categories, despite utilizing similar selection criteria.

Lastly, with regards to sampling, the majority of samples within both the Carnegie collection and the Kyoto collection were placed within fixative, and hence researchers such as O’Rahilly standardized this amongst their samples, ensuring that all embryos under scrutiny had been placed within fixative for a period of time. O’Rahilly comments on this use of fixation and highlights how it may result in a change in length, although the extent and direction of this change in embryonic length requires further studies [4].

### 8.2. Technological Advances: A Cause for Discrepancies?

As our understanding of embryology has grown, so have our technological capabilities. Compared to past methods, present-day embryonic age estimation has been rendered far more precise due to refined fertilization dating methods/techniques. This, in combination with improved ultrasonography (invented 1956), has provided a new approach to embryonic age estimation. Furthermore, ultrasonography in vivo has provided a new approach to sonoembryology, contributing to more adept measurements of embryonic length [30]. Alongside this, technological advancements such as three-dimensional ultrasound, three-dimensional reconstructions, and virtual embryoscopy all aid in providing more means to determine embryonic age and, as such, can assist in providing a more refined estimation. A perfect showcase of how these newfound techniques may play an instrumental role in the future of embryological studies can be found within recent studies conducted by Dr. M. Rousian et al. in Rotterdam. Her publications have shed light on how three-dimensional ultrasound and virtual reality are ideal for visualizing embryological structures and also how these specialized techniques can help to evaluate embryonic growth and development [31,32,33], extending to areas such as brain development, which has always been complex and challenging to study. Furthermore, Rousian et al. employed embryonic volume as a measure of embryonic growth, in addition to CRL and other previously established methods of measurement [31]. Therefore, taking into consideration the large extent to which technology has grown, these innovative techniques may indeed play a role in the formation of these recorded differences, despite being acknowledged.

### 8.3. Staging Differences: A Matter of Data Collection?

Another potential hindrance for uniform values is differing methods of data collection. Regarding embryonic length within O’Rahilly’s study, measurements were carried out with the use of calipers (measuring to 0.1 mm accurately), without any attempt to straighten the natural curvature of the embryo [4]. Additionally, accurately scaled models were utilized initially, supplanting the pre-stage 10 embryos, as up until this stage, the embryos were too small to accurately measure through the use of calipers. Within his 2010 revision, however, O’Rahilly used enlarged photographs, graphic reconstructions, and solid plaster reconstructions to measure embryonic length [24]. Despite these plaster reconstructions showcasing a relative decrease in size, proper adjustments were made to the recordings to adequately account for this. Contrary to this, Harkness obtained embryonic lengths by placing the embryos under analysis upon a 1 mm graph paper, where their measurements were then recorded under a dissecting microscope [21]. This marked difference in data collection techniques might serve as a factor behind the differences observed within embryonic length. In addition to this, throughout his revision, O’Rahilly makes his aversion towards the use of CRL clear, highlighting how the point of measurement directly above the midbrain (crown) and the definitive point of the rump was hard to determine, leading to inaccuracies. This and the inability to measure CRL in exceedingly young embryos, as these structures simply cannot be identified, serve as potential reasons behind the relatively large differences that can be observed within Harkness and O’Rahilly’s embryonic length values. Despite this, the aforementioned reasons do little to explain the differences found within embryonic ages.

### 8.4. Embryonic Diapause: A Novel Theory

Serving as a reproductive strategy present within a variety of different mammals, embryonic diapause (ED) can be defined as the temporary arrest of embryonic development. This occurs through a delayed implantation into the uterus, resulting in a dormant yet competent zygote [21]. In the absence of appropriate uterus stimulation, the metabolism of the embryo is slowed, resulting in an extension of the gestational period. Aside from its use as a protective phenomenon, little is known regarding ED, and the climatic, metabolic, and psychosocial conditions required for its occurrence are not well understood. Within animals, ED is believed to be the consequence of physiological stressors (e.g., day length), whereas, within humans, it is conversely believed to be the consequence of psychological stress [21].

Delayed implantation, as a process, has long been identified within humans from as early as 1996 and has been associated with adverse pregnancy outcomes. Should ED occur in humans, the current clinical use of LMP in the estimation of ovulation and embryonic age would be inevitably misleading and, as such, would provide a viable explanation into the variability witnessed within embryonic ages across academic publications.

### 8.5. Limitations

In our attempt to provide clarity and guidance through a review of the literature (Table 5), certain factors complicated our aim. Initially, we did find a diverse range of works from the literature. However, upon a deeper dive, it was found that the majority of current embryological publications simply defaulted to the use of O’Rahilly’s set of values from 1987, due to its esteemed stature. The reasoning for this is presumably the fact that most embryological research must be based on a pre-existing collection, as acquiring embryonic samples is ethically challenging and time-consuming. As such, researchers turn to pre-established studies regarding embryological collections, most definitively of which would be O’Rahilly’s revered study in 1987. However, the frequent use of O’Rahilly’s values did not consider the precision or accuracy of these values but is instead based upon its widespread and familiar nature.

Another potential limitation of this study would be the lack of access to certain academic works, especially those published early in the 19th–20th century (such as some of Streeter’s publications between 1873 and 1948), as these publications may be of academic importance but were not effectively covered within this review.

### 8.6. Future Perspectives

A clear basic understanding of the embryonic staging system enables a more accurate estimation of embryonic age and its associated internal and external features and, as such, helps prevent erroneous gestational age estimation, along with offering a more accurate monitoring of natural embryonic development. Despite a high level of variance across each of the academic publications, a clear overview of the current embryonic literature regarding Carnegie stages was provided, highlighting their independent differences. By providing potential factors behind these differences, alongside individual considerations, we believe we provided a first step towards a more uniform and reliable system and guidance towards a universal staging procedure. This review is helpful for clinicians and serves as a setup for further embryological research. Consequently, future research should concentrate on combining pre-existing collections and newly formed collections, whilst standardizing data collection procedures, thereby providing a gold standard for embryonic staging that extends its scope well beyond that of pre-existing academic publications.

Beyond this, a prospective study on women pregnant through in vitro fertilization (IVF) and who are willing to undergo highly detailed ultrasounds early on in their pregnancy, would help shine a light on staging with regards to embryological development. One key hurdle within embryological research is the lack of clarity with respect to embryonic age, as within normal circumstances, the exact moment of fertilization is essentially impossible to pinpoint. Therefore, such a study would allow for a precise evaluation of embryonic age and provides insights into the developmental timeline. Additionally, such a study could effectively utilize novel technologies to calculate embryonic/fetal volume [19], allowing us to disregard measures such as the CRL, which has showcased variability in its data collection methods. As such, automated fetal selection and volume measurement through the use of artificial intelligence would remove such inconsistencies and individual variation, providing embryologists with the means to attain data that would showcase a level of certainty and precision that was previously inaccessible. However, considering the power of current ultrasound equipment, such a study is complicated, or maybe even impossible to carry out, as it remains a challenge to capture early stage embryos through ultrasound.

## 9. Conclusions

The aim of this study was to clarify the presence of a gold standard of Carnegie staging amongst the various values proposed within academic publications. Through a review of the literature, our definition list, and independent sections on embryonic age and length, we strived to clarify the terminology and understanding within embryological studies on Carnegie staging. By evaluating the large discrepancies within embryonic ages and lengths and supplementing them with possible explanations, we can propose Hill’s paper from 2007 as the gold standard of embryological staging [5]. Through its multi-collection samples, alongside its access to modern technological advancements and data collection techniques, Hill avoids some of the key pitfalls present within the various other studies analyzed and, as such, should be consulted with regards to its values on embryonic length and age. Despite Hill serving as the current gold standard, we firmly believe that there is much room for improvement and that a truly accurate and applicable gold standard does not exist nationally nor internationally yet.

## Figures and Tables

**Table 1:** Overview of embryonic age (mean days after fertilization) based on Carnegie stages of human embryos, according to various publications.

Carnegie	Mean Days					
**stage**	**Heirloom collection** **(2006)** **[23]**	**O’Rahilly** **(1987)** **[4]**	**O’Rahilly** **(2010)** **[24]**	**Hill** **image** **(2018)** **[18]**	**Hill** **(2007)** **[5]**	**Harkness** **(1997)** **[21]**
1	1	1	1	1	-	-
2	2.5	2.25	2.5	3	-	-
3	4.5	4	4.5	4	-	-
4	6	5.5	6	-	-	-
5	9.5	9.5	9.5	-	-	-
6	17	13	17	-	-	-
7	19	16	19.5	16	16	-
8	23	18	23	18	18	25.5
9	25	20	26	20	20	31
10	28	22	29	22	22.5	31.2
11	29	24	29	24.5	24.5	30
12	30	26	30	28	28	35.5
13	32	28	31.5	30	30	36.2
14	33	32	34	33	33	37.9
15	36	33	36	36.5	36.5	38.2
16	39	37	38.5	39.5	39.5	40.3
17	41	41	40.5	43	43	40.8
18	44	44	43.5	46	46	41.9
19	46	47.5	46	49.5	49.5	44.3
20	49	50.5	48.5	52	52	43.9
21	51	52	50.5	53.5	53.5	45
22	53	54	53.5	56	55	50
23	56	56.5	55.5	58	58	49.9

**Table 2:** Overview of embryonic length (mm) based on Carnegie stages of human embryos, according to various publications.

Carnegie	Mean Length (mm)				
**Stage**	**Heirloom collection** **(2006)** **[23]**	**O’Rahilly** **(1987)** **[4]**	**O’Rahilly** **(2010)** **[24]**	**Hill** **(2007)** **[5]**	**Harkness** **(1997)** **[21]**
1	0.125	0.125	0.125	-	-
2	0.15	0.15	0.15	-	-
3	0.15	0.15	0.15	-	-
4	0.15	0.15	0.15	-	-
5	0.15	0.15	0.15	-	-
6	0.2	0.2	0.3	-	-
7	0.4	0.4	0.6	0.4	-
8	1.25	1.25	1.1	1.5	2.5
9	2	2	1.4	2	2.5
10	2.75	2.25	2.1	2.75	2.9
11	3.5	3.5	3.2	3.5	3.2
12	4	4	3.9	4	4.2
13	5	5	4.9	5	5.6
14	6	6	6.5	6	7.2
15	8	8	7.8	8	8.7
16	9.5	9.5	9.6	9.5	10
17	12.5	12.5	12.2	12.5	12.2
18	15	15	14.9	15	14.8
19	17	18.5	18.2	17	16.9
20	20	22	20.7	20	18.1
21	23	23	22.9	23	22.3
22	25.5	26	25.5	25.5	22.8
23	29	29	28.8	29	23.7

**Table 4:** Overview of notable embryonic collections.

Collection	Place	Number	Characteristics	Establishment
Carnegie	Washington, DC, USA	~10,000	Human histology and fixed specimens	1887
Madrid	Madrid, Spain	~120	Human histology	1935
Blechschmidt	Göttingen, Germany	100	Human histology	1950s
Kyoto	Kyoto, Japan	~44,000	Human histology and fixed specimens	1961

**Table 5:** Table of included studies.

Author	Publication Year	Title
Harkness, L.	1997	“Morphological and molecular characteristics of living human fetuses between Carnegie stages 7 and 23: developmental stages in the post-implantation embryo.”
HDBR Atlas	2010	Human Developmental Biology Resource.
Heirloom Collection	2006	Human embryo imaging and reconstruction, Library of Online media collection.
Hill, M. A.	2007	*Early Human Development.*
Hill, M. A.	2018	“Developing the Digital Kyoto Collection in Education and Research”
Jirásek, J. E.	1972	*Development of the genital system and male pseudohermaphroditism.*
Nishimura, H.; Takano, K.; Tanimura, T.; and Yasuda, M.	1968	“Normal and abnormal development of human embryos: First report of the analysis of 1213 intact embryos.”
O’Rahilly, R.; Muller, F.; and Streeter, G. L.	1987	“Developmental stages in human embryos: Including a revision of Streeter’s “Horizons” and a survey of the Carnegie collection.”
O’Rahilly, R.; and Müller, F.	2010	“Developmental Stages in Human Embryos: Revised and New Measurements.”

## Data Availability

Not applicable.
